# Improving lipid-lowering treatment and cardiovascular risk management in primary care: findings from an audit and feedback project

**DOI:** 10.3389/fphar.2026.1787952

**Published:** 2026-03-27

**Authors:** Michelangelo Rottura, Selene Francesca Anna Drago, Antonino Molonia, Viviana Maria Gianguzzo, Federica Maria Sacco, Giovanni Pallio, Natasha Irrera, Riccardo Scoglio, Sebastiano Marino, Salvatore Corrao, Giuseppe Mandraffino, Egidio Imbalzano, Vincenzo Arcoraci

**Affiliations:** 1 Department of Clinical and Experimental Medicine, University of Messina, Messina, Italy; 2 Department of Chemical, Biological, Pharmaceutical and Environmental Sciences, University of Messina, Messina, Italy; 3 Department of Biomedical and Dental Sciences and Morphological and Functional Imaging, University of Messina, Messina, Italy; 4 Italian Society of General Practice, Section Messina, Messina, Italy; 5 Dipartimento di Promozione Della Salute, Materno Infantile,Medicina Interna e Specialistica di Eccellenza “G. D’Alessandro”, PROMISE, University of Palermo, Palermo, Italy

**Keywords:** appropriateness, audit and feedback, cardiovascular risk, LDL-C target, lipid-lowering therapy, multidisciplinary approach, primary care

## Abstract

**Introduction:**

Cardiovascular diseases remain the leading cause of morbidity and mortality worldwide. Low-density lipoprotein cholesterol (LDL-C) plays a central role in atherogenesis and cardiovascular events. In real-world primary care, many patients at high and very-high cardiovascular risk (HCVR) do not achieve recommended LDL-C targets.

**Objectives:**

This study evaluated whether an audit and feedback (A&F) intervention could improve lipid management in HCVR patients managed in general practice.

**Methods:**

We conducted a retrospective–prospective observational study involving general practitioners (GPs). GPs were classified as active, participating in A&F training and feedback sessions, or controls, contributing data only. Patient-level data were extracted from electronic medical records across three 18-month observation periods (T0, T1, and T2). Active GPs received tailored performance reports and educational sessions after T0 and T1. Main outcomes included completeness of clinical data recording, LDL-C target attainment (<70 mg/dL), lipid-lowering treatment patterns, and treatment adherence.

**Results:**

Compared with baseline, active GPs showed significant improvements in the recording of LDL-C (+18.3%), HbA1c (+5.7%), body mass index (+17.1%), smoking status (+4.4%), and alcohol consumption (+14.4%). LDL-C target attainment increased from 6.1% at T0 to 14.5% at T2, exceeding the proportion observed in controls at T2 (8.7%). The use of lipid-lowering therapy among active GPs increased from 48.9% at T0 to 61.3% at T2, while high-intensity therapy increased from 9.1% to 17.6%. Treatment adherence improved over time, with a higher proportion of patients adherent to high-intensity therapy and a reduction in non-adherent low-intensity therapy.

**Conclusion:**

An audit and feedback intervention supported by a multidisciplinary team improved data completeness and lipid management in high-risk patients in the primary care setting.

## Introduction

Atherosclerotic cardiovascular disease (ASCVD) remains a major cause of morbidity and mortality worldwide, despite declining incidence and mortality rates in many European countries (Kotseva et al., 2021). Effective prevention of cardiovascular (CV) events requires coordinated, multifactorial interventions aimed at reducing modifiable risk factors and preventing disease progression. The growing prevalence of diabetes mellitus (DM) and obesity further increases ASCVD burden and complicates long-term prevention strategies (Cooney et al., 2009).

Among modifiable risk factors, Low-Density Lipoprotein Cholesterol (LDL-C) plays a pivotal role in atherogenesis (Boekholdt et al., 2014; Vallejo-Vaz et al., 2017). Retention of cholesterol-rich lipoproteins in the arterial wall is a key initiating event (Baber et al., 2015), and reductions in LDL-C consistently translate into reduced CV risk. Importantly, risk reduction is proportional to the absolute decrease in LDL-C, independent of the strategy used to achieve it (Silverman et al., 2016). Even modest LDL-C lowering is clinically meaningful, particularly in patients at high or very high CV risk (Ference et al., 2018).

International guidelines recommend high-intensity lipid-lowering strategies, typically beginning with high-potency statins (e.g., rosuvastatin or atorvastatin), adding ezetimibe when needed, and escalating to Proprotein Convertase Subtilisin/Kexin type 9 (PCSK9) inhibitors or other agents (e.g., bempedoic acid) for patients who remain above target (Mach et al., 2020; 2025). Nevertheless, target attainment remains suboptimal in real-world practice, even when effective medications are available (Rottura et al., 2021; 2022).

General Practitioners (GPs) are essential for chronic disease management (Savoy et al., 2017; Marcussen et al., 2021), including cardiovascular prevention (Rottura et al., 2021; 2023). However, implementing guideline-based care in primary care requires organizational support, systematic follow-up, and effective use of digital clinical information (Glenngård and Anell, 2021). In this context, audit and feedback (A&F) has been widely adopted to improve quality of care by comparing practice against defined standards, providing actionable performance feedback, and supporting change through education. Despite its widespread use, the magnitude of clinical impact varies across settings, and evidence on effectiveness in improving LDL-C target attainment in primary care remains limited (Jamtvedt et al., 2003; Piacentini et al., 2005; Corrao et al., 2009; Arcoraci et al., 2014).

This study aimed to evaluate whether an A&F program, delivered through a multidisciplinary collaboration (including clinical pharmacologists, cardiologists, and general practitioners), could improve the monitoring and pharmacological management of patients at high or very high CV risk in general practice. We hypothesized that structured feedback and tailored educational sessions would increase data completeness, improve prescribing patterns, enhance adherence, and ultimately increase LDL-C target achievement.

## Materials and methods

### Study design and setting

We conducted a retrospective–prospective observational study within a structured A&F quality improvement program implemented in primary care. The project involved 13 GPs operating in the province of Messina (Sicily, Italy) affiliated with ARM (Audit & Research Messina) group in collaboration with the Department of Pharmacology of the University Hospital “G. Martino” in Messina, which coordinated the audit process and feedback activities.

All members of the ARM network were invited to participate in the A&F program and were informed about the study design. Recruitment occurred on a voluntary basis. Gps who did not attend the educational program were included in control group

All participating physicians operated within the same provincial healthcare system and under the same regional organizational and prescribing framework. Practice characteristics were broadly comparable, as all GPs belonged to the same professional network and worked within a geographically homogeneous setting.

Participating GPs were categorized according to their level of engagement in the A&F intervention:

- Active GPs (n = 9): participated in all A&F educational and informational sessions.
- Control GPs (n = 4): did not participate in any sessions; their data served as a comparison group.

This design enabled both within-group longitudinal analyses and between-group comparisons, thereby strengthening the evaluation of the intervention’s impact.

### Data sources and observation periods

Patient-level data were obtained from GPs’ electronic medical records (EMRs), which systematically collect demographic characteristics, clinical parameters, laboratory results, and pharmacological prescriptions. Data were analyzed across three predefined observation periods:

- Pre-audit (T0): 01/01/2018–06/30/2019 (extracted on 06/30/2019)
- Post-audit (T1): 07/01/2019–12/31/2020 (extracted on 12/31/2020)
- Implementation (T2): 01/01/2021–06/30/2022 (extracted on 06/30/2022)

Physicians autonomously chose whether to participate as active GPs, attending the educational and feedback sessions, or as control GPs, contributing anonymized EMR data only at the final observation period (T2). No randomization or predefined allocation criteria were applied; group classification was entirely based on voluntary participation and level of engagement in the intervention.

These observation periods were chosen to represent the baseline phase, the immediate post-intervention phase, and a longer-term implementation phase, thereby enabling the assessment of both short-term effects, such as rapid changes in documentation and prescribing behavior following feedback, and medium-term effects, which reflect the sustainability of these improvements and the integration of guideline-based practices into routine care.

### Audit and feedback intervention

The A&F intervention was implemented as a multistep, iterative process combining systematic data auditing, structured feedback delivery, and targeted educational support. Feedback sessions were provided exclusively to active GPs at three predefined time points, each corresponding to the end of an observation period. These sessions included individualized performance reports, benchmark comparisons against recommended standards, and tailored educational materials designed to facilitate interpretation of results and promote evidence-based practice:

- 06/30/2019: aggregate results for T0 were presented, including individualized reports summarizing lipid management and LDL-C target attainment.
- 12/31/2020: comparative results for T0 vs. T1 were discussed, followed by updated feedback and tailored materials.
- 06/30/2022: results for T2 were reviewed and compared with T0 and T1; performance of active vs. control GPs was also discussed.

Feedback reports included aggregate data, individualized performance indicators, and benchmark comparisons against recommended standards, encouraging reflection on prescribing behavior and overall clinical management. Educational activities were delivered with the support of clinical pharmacologists and cardiologists, fostering a multidisciplinary approach.

**FIGURE 1:**
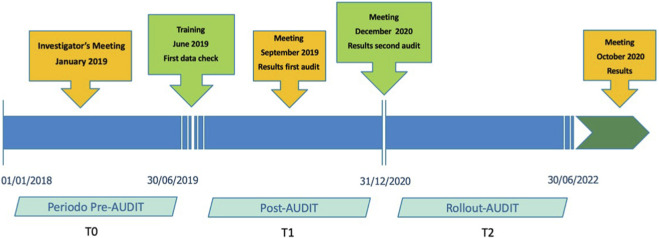
Representation of the periods and training events conducted during the study. Control GPs provided data only at T2; therefore, longitudinal data for T0 and T1 were not available for this group.

Control GPs did not receive any feedback or educational intervention during the study period; their EMR data were collected in aggregate form during the T2 phase and used exclusively for comparative analyses (Figure 1).

### Dataset and study population

The dataset included anonymized patient-level information extracted from electronic medical records. Collected variables comprised demographic characteristics (encrypted patient identifier, age, and sex), lifestyle factors (weight, height, body mass index, smoking status, and alcohol consumption), and clinical and laboratory measures, including blood pressure, low-density lipoprotein cholesterol (LDL-C), total cholesterol, high-density lipoprotein cholesterol (HDL-C), triglycerides, fasting glucose, and glycated hemoglobin (HbA1c).

Information on comorbidities was retrieved using International Classification of Diseases, Ninth Revision (ICD-9) codes, while pharmacological treatments were identified through Anatomical Therapeutic Chemical (ATC) codes and drug specialties. Data on specialist referrals, including cardiology and other outpatient visits, were also collected.

Patient anonymity was ensured through the use of encrypted identification codes. The study protocol was approved by the local Ethics Committee of the Messina University Hospital (protocol no. 0010280/2020; coordinating center) and was conducted in accordance with the principles of the Declaration of Helsinki.

According to European Society of Cardiology (ESC) criteria, patients were classified as having high or very high cardiovascular risk if they met at least one of the following conditions: familial dyslipidemia (ICD-9 272*); diabetes with complications (ICD-9 250.1–250.9); diabetes (ICD-9 250*) associated with hypertension (ICD-9 401*–405*), obesity (ICD-9 278*), or dyslipidemia (ICD-9 272*); diabetes in current smokers; atherosclerosis (ICD-9 440*, 444*); chronic kidney disease (ICD-9 585*); ischemic heart disease (ICD-9 410*–414*); cerebrovascular disease (ICD-9 430*–438*); or severely elevated lipid levels (LDL-C ≥190 mg/dL or total cholesterol ≥310 mg/dL).

### Outcomes and definitions

Data completeness was assessed as the proportion of patients with a recorded value for each clinical or laboratory variable, including LDL-C and HbA1c. LDL-C levels were categorized as <70 mg/dL, 70–99 mg/dL, 100–159 mg/dL, ≥160 mg/dL, or missing. LDL-C target attainment was defined as LDL-C <70 mg/dL. In addition, the proportion of patients achieving LDL-C <55 mg/dL was evaluated as a secondary descriptive analysis.

Lipid-lowering therapy included statins and/or ezetimibe. High-intensity therapy was defined as rosuvastatin >10 mg or atorvastatin >20 mg, or rosuvastatin/atorvastatin/simvastatin combined with ezetimibe, consistent with ESC definitions used in the study.

Medication adherence was assessed using the Medication Possession Ratio (MPR), defined as the proportion of days covered by dispensed medication within each period. Patients were categorized as:

- Adherent, high intensity: high-intensity therapy and MPR ≥80%
- Adherent, low intensity: low-intensity therapy and MPR ≥80%
- Non-adherent, high intensity: high-intensity therapy and MPR <80%
- Non-adherent, low intensity: low-intensity therapy and MPR <80%

### Statistical analysis

We performed descriptive analyses of clinical and demographic characteristics for each period. Frequencies and proportions were calculated for categorical variables, and medians with interquartile ranges (Q1–Q3) were used for continuous variables such as age and LDL-C. The Kolmogorov–Smirnov test was used to assess normality. Because variables were non-normally distributed, non-parametric approaches were adopted.

Univariate and multivariable logistic regression models were used to identify predictors of lipid-lowering therapy and predictors of LDL-C target achievement. Results are reported as odds ratios (OR) with 95% confidence intervals (CI). Statistical significance was defined as p < 0.05. Analyses were performed using SPSS version 23.0 (Statistical Package for the Social Sciences, IBM Corp., Armonk, NY, USA).

## Results

### Population

**FIGURE 2:**
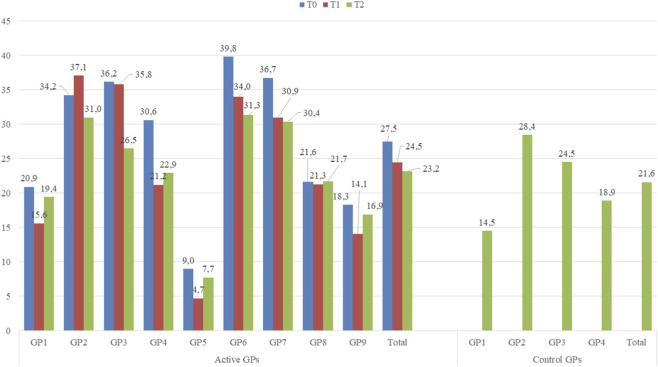
Prevalence of H/VHCVr patients stratified by GP in different follow-up periods.

Among all patients managed by GPs in each period, high/very-high CV risk patients represented 3,093 (27.5%) in T0, 2,978 (24.5%) in T1, and 2,914 (23.2%) in T2. In the control group, 953 H/VHCVr patients (21.6%) were identified in T2. The number of H/VHCVr patients varied across individual GPs (Figure 2).

### Completeness of clinical recording

Active GPs showed significant improvements in the recording of key risk-related variables over time, including LDL-C (+18.3% from T0 to T2), glycosylated hemoglobin (HbA1c) (+5.7%), body mass index (BMI) (+17.1%), smoking status (+4.4%), and alcohol consumption (+14.4%). Most other laboratory variables remained stable, while blood pressure recording decreased during follow-up (−10.2%). During T2, active GPs generally recorded a higher proportion of clinical variables compared with control GPs, with exceptions noted for BMI and alcohol consumption (Table 1).

**TABLE 1:** Records of laboratory values and lifestyle habits stratified by observation period of active and control GPs.

​	Active GPs			Control GPs
	T0	T1	T2	T2
Patients	3,093 (%)	2,978 (%)	2,914 (%)	953 (%)
Age; years [median (Q1-Q3)]	70 (60–79)	73 (63–81)^a^ ^,^ ^c^	71 (62–79)^b^	70 (60–78)
LDL-C [median (Q1-Q3)]	118 (90–114)^a^ ^,^ ^b^	105 (81–133)^c^	102 (77–130)	103 (80–133)
Recorded value; N (%)				
Blood pressure	2,337 (75,6)^a^ ^,^ ^b^	1992 (66,9)	1905 (65,4)^d^	409 (42,9)
Glycemia	2,697 (87,2)^a^	2,382 (80,0)	2,549 (87,5)^c^ ^,^ ^d^	575 (60,3)
HbA1c	1,436 (46,4)^a^	1,068 (35,9)	1,518 (52,1)^b^ ^,^ ^c^ ^,^ ^d^	306 (32,1)
LDL-C	2032 (65,7)	2,303 (77,3)^a^	2,457 (84,3)^b^ ^,^ ^c^ ^,^ ^d^	598 (62,7)
Total cholesterol	2,763 (89,3)^a^ ^,^ ^b^	2,420 (81,3)	2,544 (87,3)^c^ ^,^ ^d^	391 (41,0)
HDL-C	2,692 (85,0)^a^	2,339 (78,5)	2,475 (84,9)^c^ ^,^ ^d^	379 (39,8)
Triglycerides	2,751 (88,9)^a^	2,419 (81,2)	2,550 (87,5)^c^ ^,^ ^d^	403 (42,3)
BMI	1,296 (41,9)	1,540 (51,7)^a^	1783 (61,2)^b^ ^,^ ^c^	592 (62,1)
Smoking	1,276 (41,3)	1,186 (39,8)	1,409 (48,4)^b^ ^,^ ^c^	453 (45,5)
Consumption alcohol	699 (22,6)	658 (22,1)	1,079 (37,0)^b^ ^,^ ^c^	416 (43,7)^^^

^a^ T0 vs. T1; p < 0.05.

^b^ T0 vs. T2; p < 0.05.

^c^ T1 vs. T2; p < 0.05.

^d^ T2 Active GPs, vs. T2 Control GPs.

BMI, body mass index; HDL-C, High-density lipoproteins cholesterol; HbA1c, glycated hemoglobin; GPs, General practitioners; LDL-C, Low-density lipoprotein cholesterol; Q1-Q3, first and third quartile.

### LDL-C levels and target attainment

Median LDL-C values decreased across periods among patients managed by active GPs (T0: 118 mg/dL; T1: 105 mg/dL; T2: 102 mg/dL). The proportion of patients achieving LDL-C <70 mg/dL increased from 6.1% in T0 to 14.5% in T2. In contrast, control GPs achieved a lower target attainment in T2 (8.7%). The proportion of patients achieving LDL-C <55 mg/dL increased from 2.3% at T0 to 3.9% at T1 and 5.4% at T2 among active GPs, while 2.2% of patients managed by control GPs achieved this threshold at T2. The proportion of patients with missing LDL-C values decreased over time among active GPs, suggesting improved monitoring (Table 2).

**TABLE 2:** Prevalence of H/VHCVr patients in LDL-C stages stratified by follow-up periods of active and control GPs.

​	Active GPs			Control GPs
LDL-C stage	T0	T1	T2	T2
	N = 3,093 (%)	N = 2,978 (%)	N = 2,914 (%)	N = 953 (%)
<70 mg/dL	188 (6,1)	321 (10,8)	423 (14,5)	83 (8,7)
70–99 mg/dL	488 (15,8)	706 (23,7)	750 (25,7)	190 (19,9)
100–159 mg/dL	1,087 (35,1)	1,016 (34,1)	1,051 (36,1)	259 (27,2)
≥160 mg/dL	278 (9,0)	260 (8,7)	233 (8,0)	66 (6,9)
Missing mg/dL	1,053 (34,0)	675 (22,7)	457 (15,7)	355 (37,3)

GPs, General practitioners; LDL-C, Low-density lipoprotein cholesterol.

### Lipid-lowering therapy and adherence

Among active GPs, lipid-lowering therapy use increased from 48.9% (T0) to 54.6% (T1) and 61.3% (T2). Statin prescriptions increased accordingly. Use of high-intensity therapy rose from 9.1% (T0) to 13.3% (T1) and 17.6% (T2). Adherence improved substantially across periods: the proportion of patients adherent to high-intensity therapy increased from 2.4% to 14.2%, and non-adherent low-intensity therapy decreased from 34.0% to 12.6% (Table 3).

**TABLE 3:** Lipid-lowering treatment of H/VHCVr patients stratified by active and control GP follow-up periods.

​	Active GPs			Control GPs
​	T0	T1	T2	T2
	N = 3,093 (%)	N = 2,978 (%)	N = 2,914 (%)	N = 953 (%)
Lipid-lowering therapy	1,512 (48,9)	1,626 (54,6)^a^	1786 (61,3)^b^ ^,^ ^c^	596 (62,5)
Statin	1,422 (46,0)	1,516 (50,9)^a^	1,622 (55,7)^b^ ^,^ ^c^	506 (53,1)
Ezetimibe	83 (2,7)	121 (4,1)^a^	143 (4,9)^b^	67 (7,0)^d^
Statin and ezetimibe	84 (2,7)	141 (4,7)^a^	260 (8,9)^b^ ^,^ ^c^	127 (13,3)^d^
High intensity therapy	282 (9,1)	395 (13,3)^a^	514 (17,6)^b^ ^,^ ^c^	201 (21,1)^d^
Adherent and HIT	74 (2,4)	319 (10,7)^a^	414 (14,2)^b^ ^,^ ^c^	160 (16,8)
Adherent and LIT	177 (5,7)	823 (27,6)^a^	905 (31,1)^b^ ^,^ ^c^	300 (31,5)
No adherent and HIT	208 (6,7)^a^ ^,^ ^b^	76 (2,6)	100 (3,4)^c^	41 (4,3)
No adherent and LIT	1,053 (34,0)^a^ ^,^ ^b^	408 (13,7)	367 (12,6)^d^	95 (10,0)

^a^ T0 vs. T1; p < 0.05.

^b^ T0 vs. T2; p < 0.05.

^c^ T1 vs. T2; p < 0.05.

^d^ T2 Active GPs, vs. T2 Control GPs.

GPs, General practitioners; HIT, high intensity therapy; LIT, low intensity therapy.

### Predictors of lipid-lowering therapy and LDL-C target attainment

Lipid-lowering therapy was more likely among patients with dyslipidemia (OR 6.05; 95% CI 4.97–7.36), hypertension (OR 1.49; 95% CI 1.19–1.86), ischemic heart disease (OR 1.92; 95% CI 1.56–2.35), diabetes mellitus (OR 1.31; 95% CI 1.10–1.58), and among patients with cardiology visits (OR 1.60; 95% CI 1.33–1.94). Lower likelihood of treatment was observed among patients with chronic respiratory diseases and mood disorders, and with increasing number of concomitant conditions (Table 4).

**TABLE 4:** Probability to be treated with Lipid-lowering drugs.

​	Univariate OR [95% CI]	P-value	Multivariate OR [95% CI]	P-value
Age (years)	1.02 (1.01–1.03)	<0.01	1.01 (0.99–1.01)	0.14
Gender (F)	0.87 (0.75–1.01)	0.07	1.09 (0.91–1.30)	0.37
Smocking	0.91 (0.70–1.17)	0.44	​	​
Alcohol	1.25 (0.92–1.72)	0.16	​	​
Comorbidity				
Dyslipidemias	4.34 (3.69–5.09)	<0.01	6.05 (4.97–7.36)	<0.01
Atherosclerosis	1.27 (1.05–1.54)	<0.01	1.24 (0.97–1.58)	0.09
Hypertension	1.87 (1.57–2.22)	<0.01	1.49 (1.19–1.86)	<0.01
Obesity	0.87 (0.71–1.07)	0.19	1.19 (0.93–1.52)	0.17
Ischemic heart disease	1.93 (1.63–2.29)	<0.01	1.92 (1.56–2.35)	<0.01
Heart failure	1.05 (0.75–1.46)	0.77	0.97 (0.66–1.40)	0.85
Cerebrovascular disease	0.84 (0.73–0.98)	0.03	1.21 (0.98–1.50)	0.08
Diabetes	1.502 (1.29–1.75)	<0.01	1.31 (1.10–1.58)	<0.01
Chronic kidney disease	1.56 (1.24–1.97)	<0.01	1.05 (0.77–1.43)	0.74
Arthritis and arthrosis	0.99 (0.85–1.15)	0.90	1.04 (0.84–1.28)	0.74
Mood disorders	0.73 (0.63–0.85)	<0.01	0.63 (0.51–0.77)	<0.01
Osteoporosis	1.04 (0.89–1.23)	0.61	1.13 (0.93–1.37)	0.23
Neoplasm	1.03 (0.82–1.28)	0.83	​	​
Chronic respiratory diseases	0.76 (0.65–0.89)	<0.01	0.69 (0.56–0.85)	<0.01
Number of diseases	1.09 (1.06–1.12)	<0.01	0.90 (0.85–0.95)	<0.01
LDL target	3.78 (2.84–5.04)	<0.01	​	​
Specialist counselling	2.01 (1.71–2.36)	<0.01	1.60 (1.33–1.94)	<0.01
N. Prescription	1.02 (1.01–1.02)	<0.01	1.02 (1.01–1.02)	<0.01
N. Different molecules	1.06 (1.05–1.07)	<0.01	0.99 (0.97–1.01)	0.480

CI, confidence interval; F, female; LDL-C, Low-density lipoprotein cholesterol; OR, odd ratio.

LDL-C target achievement was strongly associated with treatment intensity and adherence. Compared with non-adherent low-intensity therapy, adherence to high-intensity therapy was associated with markedly higher odds of achieving LDL-C targets. Nevertheless, a substantial proportion of patients remained above target even when adherent to high-intensity therapy (Table 5).

**TABLE 5:** Type of lipid-lowering treatment and LDL-C categories.

​	<70 mg/dL	70–99 mg/dL	100–159 mg/dL	≥160 mg/dL	Missing	OR (IC95%) LDL target
N. patients	362 (%)	555 (%)	539 (%)	148 (%)	182 (%)	​
Adherent to HIT	126 (34,8)	126 (22,7)	89 (16,5)	19 (12,8)	54 (29,7)	3,43 (2,35–5,03)
Adherent to LIT	172 (47,5)	305 (55,0)	281 (52,1)	67 (45,3)	80 (44,0)	1,68 (1,18–2,40)
No adherent to HIT	19 (5,2)	21 (3,8)	33 (6,1)	14 (9,5)	13 (7,1)	1,78 (0,98–3,24)
No adherent to LIT	45 (12,4)	103 (18,6)	136 (25,2)	48 (32,4)	35 (19,2)	#Ref

CI, confidence interval; HIT, high intensity therapy; LDL-C, Low-density lipoprotein cholesterol; LIT, low intensity therapy; OR, odd ratio.

Moreover, in multivariable analysis, achieving the LDL‐C target was positively associated with older age (OR 1.01 per year, 95% CI 1.00–1.02), a higher number of non lipid lowering prescriptions (OR 1.01, 95% CI 1.00–1.01), and treatment adherence—particularly to high intensity regimens (OR 5.50, 95% CI 3.07–9.86). Conversely, male sex (OR 0.42, 95% CI 0.33–0.53) and receiving multiple lipid lowering agents (OR 0.72, 95% CI 0.53–0.98) were associated with lower odds of target attainment (Table 6).

**TABLE 6:** Predictive factors for achieving the lipid target in patients at high and very high cardiovascular risk (LDL-C<70 mg/dL).

​	Univariate OR [95% CI]	P-value	Multivariate OR [95% CI]	P-value
Age (years)	​	<0.01	1.01 (1.00–1.02)	0.03
Gender (M)	0.44 (0.35–0.41)	<0.01	0.42 (0.33–0.53)	<0.01
Smocking	0.97 (0.70–1.34)	0.84	​	​
Alcohol	1,37 (0,92–2,04)	0.12	​	​
Number of diseases	1,03 (0,99–1,07)	0.17	0,97 (0,93–1,02)	0.25
Specialist counselling	1,78 (1,37–2,33)	<0.01	1,15 (0,87–1,53)	0.33
N. of molecules	1.04 (1.03–1.06)	<0.01	1,14 (0,86–1,53)	0.58
N. of prescription	1.01 (1.01–1.01)	<0.01	1.01 (1.00–1.01)	<0.01
N. Lipid-lowering molecules	1.91 (1.64–2.21)	<0.01	0,72 (0,53–0,98)	0.03
N. Lipid-lowering prescription	1.12 (1.09–1.14)	<0.01	1.05 (1.02–1.08)	<0.01
Lipid-lowering therapy	3,78 (2,84–5.04)	<0.01	​	​
No lipid-lowering therapy	#Ref	<0.01	#Ref	​
No adherent to LIT	2,24 (1,33–3,78)	<0.01	2,24 (1,33–3,78)	0.03
No adherent to HIT	2,88 (1,80–4,59)	<0.01	2,88 (1,80–4,59)	<0.01
Adherent to LIT	3,79 (1,85–7,75)	<0.01	3,79 (1,85–7,75)	<0.01
Adherent to HIT	6,99 (3,07–9,86)	<0.01	5,50 (3,07–9,86)	<0.01

CI, confidence interval; HIT, high intensity therapy; LDL-C, Low-density lipoprotein cholesterol; LIT, low intensity therapy; M, male; OR, odd ratio.

## Discussion

Cardiovascular diseases (CVD) remain the leading cause of morbidity and mortality worldwide, accounting for approximately one-third of all deaths (Joseph et al., 2017) The risk of developing these conditions is strongly associated with both modifiable and non-modifiable factors. Among these, LDL-C plays a pivotal role: robust evidence demonstrates that elevated LDL-C concentrations are directly proportional to atherosclerotic plaque progression and the incidence of ASCVD events (Ference et al., 2017). However, cardiovascular risk is also influenced by other clinical parameters (blood pressure, glycemic profile, BMI, renal function) and lifestyle factors such as smoking and alcohol consumption (Teo and Rafiq, 2021). For this reason, a multidisciplinary approach involving different healthcare professionals is essential to optimize patient management, particularly in primary care settings.

Our study shows that an A&F program, implemented in collaboration between GPs and specialists (clinical pharmacologists and cardiologists), can significantly improve the management of patients at H/VHCVr. One of the main achievements was increasing GPs’ awareness of their patients’ clinical profiles. Before the intervention, the recording of key parameters was limited: only 66% of patients had LDL-C documented, and less than half had HbA1c, BMI, or lifestyle habits recorded. This gap was not due to inadequate care but to inertia in electronic data entry. Digitalization of health information is crucial for improving data availability and completeness, thereby enhancing chronic disease prevention (Kruse et al., 2018).

Following the A&F intervention, a significant improvement in data recording was observed: LDL-C (+18.3%), BMI (+17.1%), HbA1c (+5.7%), alcohol consumption (+14.4%), and smoking status (+4.4%). These changes enabled more accurate risk stratification and better clinical decision-making. Our findings on the enhanced documentation of cardiovascular risk factors and clinical profiles align with existing evidence supporting the effectiveness of A&F interventions. A comprehensive Cochrane review of 140 randomized controlled trials reported that A&F generally produces modest but clinically relevant improvements in professional practice, with the magnitude of effect varying according to context, baseline performance, and delivery strategies. The underlying rationale for these interventions lies in providing clinicians with structured performance data benchmarked against explicit standards, thereby fostering awareness and motivating behavior change (Tuti et al., 2017).

This mechanism is consistent with behavioral theories such as Feedback Intervention Theory, which emphasize the role of comparative performance information in driving improvement. Furthermore, experimental and field studies have demonstrated that feedback reports combined with benchmark comparisons can positively influence health professionals’ intentions and actions, highlighting a key pathway through which A&F promotes sustained practice change (Gude et al., 2017).

In our study, these principles translated into tangible outcomes: after receiving individualized feedback and benchmark comparisons, general practitioners significantly improved the completeness of clinical recording (e.g., LDL-C +18.3%, BMI +17.1%) and optimized prescribing patterns, including a marked increase in high-intensity lipid-lowering therapy and adherence rates. These changes illustrate how structured feedback and educational support can activate behavior change pathways, ultimately enhancing cardiovascular risk management in primary care.

Indeed, Educational support amplified the impact of feedback by converting awareness into practice change. Without these sessions, feedback alone might have had limited effect. The structured learning environment enabled GPs to adopt evidence-based interventions, leading to better LDL-C monitoring and more appropriate lipid-lowering therapy.

International guidelines recommend high-intensity lipid-lowering therapy to reduce LDL-C by at least 50% and achieve targets below 70 mg/dL (Mach et al., 2020; 2025). However, real-world practice often shows poor adherence to these recommendations (Rottura et al., 2021; Arcoraci et al., 2022; 2024) and only a small proportion of patients with H/VHCVr are appropriately treated with lipid-lowering drugs (Guglielmi et al., 2017; Rottura et al., 2022). In our study, during the pre-audit phase, fewer than half of H/VHCVr patients were treated with lipid-lowering drugs, and only 2.4% were adherent to high-intensity therapy (MPR ≥80%). After the intervention, adherence improved markedly: patients on high-intensity therapy increased to 14.2%, while non-adherent patients on low-intensity therapy decreased from 34% to 12.6%.

Despite these improvements, 65% of patients adherent to high-intensity therapy still failed to achieve LDL-C targets, highlighting the need for additional strategies, including the use of innovative agents such as PCSK9 inhibitors or bempedoic acid (Moriarty et al., 2014; Langslet et al., 2015; Melendez et al., 2017; Cicala et al., 2024).

When contemporary ESC/EAS 2025 LDL-C goal for very-high-risk patients (<55 mg/dL) was adopted (Mach et al., 2025), target attainment appears even more limited. In our cohort, only 5.4% of patients managed by active GPs achieved LDL-C <55 mg/dL at T2, despite the observed improvement after the audit and feedback intervention. These findings suggest that the majority of H/VHCVr patients remain far from optimal lipid control according to current standards. Because high- or very-high-risk patients could not be reliably characterized within our dataset, we adopted the <70 mg/dL threshold for primary analyses; however, this approach surely overestimated of target in light of contemporary guideline.

Taken together, these data reinforce the need for earlier treatment intensification, broader implementation of combination therapy, and timely referral for advanced lipid-lowering strategies in real-world primary care.

Accordingly with previous research (Barbieri et al., 2020; Rottura et al., 2021; 2022) our results indicates that patients referred for specialist evaluation and those demonstrating higher treatment adherence are significantly more likely to receive appropriate prescriptions. Evidence also supports the importance of collaborative care models involving general practitioners and clinical specialists to optimize cardiovascular risk management (Scalvini et al., 2011). Moreover, structured follow-up after specialist referral has been shown to enhance patient adherence and improve overall treatment outcomes (De Luca et al., 2005).

The A&F process proved effective in enhancing data recording and prescribing appropriateness. Nevertheless, some patients remained untreated or inadequately controlled, underscoring the need for complementary strategies such as telemedicine, digital reminders, and stronger integration between GPs and specialists. Furthermore, implementing standardized cardiovascular risk assessment tools could improve patient engagement and adherence. For patients who do not achieve lipid targets despite high-intensity therapy, referral for advanced treatments (PCSK9 inhibitors, inclisiran) is essential, as these agents have demonstrated substantial efficacy and safety.

## Strengths and limitations

This study has several strengths. It was conducted in a real-world primary care setting, reflecting routine clinical practice and providing pragmatic evidence on lipid management in patients at high and very-high cardiovascular risk. The inclusion of a large cohort of patients enhances the clinical relevance of the findings.

The A&F intervention was implemented within a multidisciplinary framework involving general practitioners, clinical pharmacologists, and cardiologists, promoting integration between primary and specialist care. The longitudinal design among active GPs (T0–T2) enabled evaluation of both short-term and medium-term changes in documentation, prescribing patterns, treatment intensity, adherence, and LDL-C target attainment.

Outcomes were based on objective EMR data, reflecting actual prescribing behavior and laboratory monitoring in routine care. In addition to LDL-C target attainment, the study assessed treatment intensity and adherence, providing a comprehensive evaluation of lipid-lowering management.

Several limitations should be acknowledged. Participation in the A&F program was voluntary and no randomization was performed. Physicians autonomously chose whether to participate as active or control GPs, introducing a potential bias, as those engaging in the intervention may have had higher baseline motivation or interest in cardiovascular prevention.

Control GPs provided data only at T2; therefore, longitudinal comparisons were limited to active GPs, and between-group analysis was restricted to cross-sectional comparison at T2. This design limits causal inference and does not fully account for potential secular trends in clinical practice.

The intervention did not include structured qualitative surveys or formal pre- and post-intervention assessment of guideline knowledge. Although improvements were observed in prescribing patterns and LDL-C control, changes in physicians’ knowledge or behavioral mechanisms cannot be directly quantified.

Clinical outcomes such as incident ASCVD events or revascularization procedures were not predefined or systematically evaluated. The study focused on process and prescribing indicators rather than adjudicated hard cardiovascular endpoints; therefore, no direct conclusions can be drawn regarding long-term cardiovascular event reduction.

Patients at high and very-high cardiovascular risk were analyzed as a combined category due to limitations in EMR-based risk stratification. Consequently, LDL-C target attainment was primarily evaluated using the <70 mg/dL threshold. When applying the contemporary ESC/EAS target for very-high-risk patients (<55 mg/dL), goal attainment was substantially lower, suggesting that lipid control may be overestimated when interpreted according to current standards.

Finally, the study was conducted within a single provincial healthcare setting, which may limit generalizability to other regions with different organizational structures or prescribing policies.

## Conclusion

This study highlights key challenges in managing patients at high and very high cardiovascular risk in primary care. Incomplete data collection and difficulty in identifying these patients often lead to suboptimal treatment and underuse of lipid-lowering therapies recommended by international guidelines. The audit and feedback program effectively addressed these gaps, improving data recording, risk stratification, and adherence to high-intensity lipid-lowering therapy. Although the proportion of patients achieving LDL-C targets remains low, the observed improvements confirm the potential of structured A&F interventions to enhance cardiovascular risk management. Scaling this model to other healthcare settings, combined with digital health solutions and multidisciplinary collaboration, could further strengthen prevention strategies and improve long-term outcomes.

## Data Availability

The raw data supporting the conclusions of this article will be made available by the authors, without undue reservation.
